# Demand for heroin in rats: effects of non-drug alternative substitutes and complements

**DOI:** 10.1038/s41386-025-02127-x

**Published:** 2025-05-09

**Authors:** David N. Kearns, Toni Bird, Emma M. Pilz, Kevin Chavez-Lopez, Felipe Rego, Alan Silberberg

**Affiliations:** American University, Psychology Department, Washington, DC USA

**Keywords:** Addiction, Psychology

## Abstract

Given the central role of economic demand for drugs in substance use disorder, identifying factors that increase or decrease drug value is of high importance. The present study investigated how the concurrent availability of non-drug reinforcers previously shown to be heroin substitutes or complements affected demand for heroin in rats. In Exp. 1, three groups of rats pressed one lever for intravenous heroin infusions on a series of prices that increased over sessions. The groups differed with respect to what they received for pressing a second lever: timeout-from-avoidance (TOA) reinforcers, heroin at a constant low price, or no programmed consequences. The essential value of heroin was significantly reduced in the groups that had either TOA or low-price heroin available on the second lever. Additionally, as the price of heroin on the first lever increased, consumption of TOA reinforcers or low-price heroin infusions increased, confirming that these were substitutes for expensive heroin. In Exp. 2, two groups of rats pressed one lever for heroin infusions on a series of increasing prices. One group could press a second lever for saccharin reinforcers at a constant low price. Concurrent saccharin availability increased demand for heroin in female rats, but not in male rats. Exp. 3 compared demand for saccharin in groups that had or did not have concurrent access to low-price heroin. Concurrent heroin availability caused an increase in estimated saccharin consumption at no cost (*Q*_0_), but did not affect elasticity of demand for saccharin. The outcome of Exp. 1 suggests that non-drug means of reducing pain or stress can weaken demand for opioids. Exp. 2 and 3 show that, in contrast, availability of some non-drug alternatives can increase demand for heroin in female rats and that heroin can increase consumption of non-drug alternatives at low price. Overall, these results show, consistent with the contextualized reinforcer pathology model, that opioid value depends on the broader behavioral economic context in which opioids and non-drug alternatives are available.

## Introduction

The behavioral economic concept of essential value (EV), which reflects the degree to which demand for a reinforcer is inelastic, is increasingly recognized as having a central role in addiction. High drug EV has been found to be associated with substance use disorder symptoms in humans [1–5] as well as poorer treatment outcomes [6, 7]. Work with animal models has also found that drug EV is associated with addiction-like behavior [8, 9]. Given the link between drug EV and addiction, identifying variables that increase or decrease drug EV could help understand factors that contribute to or guard against addiction.

According to behavioral economics, elasticity of demand for a good critically depends on the availability of substitutes [10]. As the price of a good increases, demand for it should fall more rapidly when low-cost substitutes are available. An individual should be less willing to pay increasingly high prices for a good when another good that serves the same function can be more easily obtained. In a previous study, it was found that timeout-from-avoidance (TOA), i.e., a brief period of safety from footshock [11], was a substitute for heroin in rats [12]. It is therefore hypothesized that heroin EV would be reduced by making low-cost TOA available as an alternative to heroin. Experiment 1 tested this hypothesis.

If the availability of substitutes should reduce heroin EV, then availability of complements might be expected to increase heroin EV. Complements are goods that individuals prefer to consume together, in a relatively fixed proportion. If one member of a complement pair is available at low cost, then having that good could make the individual more willing to continue to pay for the other member of the pair as its price increases. Beasley et al. [12] found in a previous study that heroin and saccharin were complements in rats. It was therefore hypothesized that heroin EV would be higher when low-cost saccharin was available than when it was not. Experiment 2 tested this hypothesis. Experiment 3 tested the symmetrical hypothesis that saccharin EV would be higher when low-cost heroin was available than when it was not.

## Methods

### Subjects

Female and male Long-Evans rats were purchased from Envigo (Livermore, CA). A total of 129 rats began procedures, but 36 of these were lost before producing usable data due to various reasons (e.g., unsuccessful surgery, catheter problems, illness, failure to acquire lever pressing, etc.). Of the remaining 93 rats, data from 9 rats (four from Exp. 1, three from Exp. 2, and two from Exp. 3) were excluded because they met Stein et al.’s [13] criteria for nonsystematic demand curve data (using the original criteria values suggested by the authors). These rats’ data are included in the tables provided in the Supplementary Materials. Rats were approximately 2.5–4 months old at the start of training. Throughout experiments, rats were treated in accordance with the Guide for the Care and Use of Laboratory Animals [14], and all procedures were approved by American University’s Institutional Animal Care and Use Committee.

### Apparatus

Training took place in Med Associates (St. Albans, VT) operant chambers that had three retractable levers on the front wall. See Supplemental Methods for more apparatus details.

### Procedure

*Exp. 1: Effects of a concurrently available TOA or heroin alternative on demand for heroin*

There were three groups in Exp. 1. All groups first learned to press the middle lever to avoid footshock (~0.4 mA, 0.5 s) on a free-operant avoidance procedure with a 35-s response-shock interval and a 5-s shock-shock interval (see Supplementary Methods for details) before having surgery to implant a jugular vein catheter (see Surgery in Supplemental Methods). After recovery from surgery, rats were assigned to either the No Alternative group, the TOA alternative group, or the Heroin Alternative group. Rats were then trained in alternating sessions to press the right lever for 0.03 mg/kg heroin infusions and to press the left lever for, depending on group, 120-s TOA periods signaled by illumination of the left-lever cue-light, termination of white noise, and retraction of levers (TOA Alternative group), 0.03 mg/kg heroin infusions (Heroin Alternative group), or no programmed consequences (No Alternative group). During these lever press acquisition sessions, the avoidance contingency remained in effect on the middle lever at all times except for rats in the TOA Alternative group when they earned a TOA reinforcer.

Once rats learned to press all three levers, the demand procedure began. Each 2-h session began with a 5-min avoidance warm-up period where only the middle lever was inserted and the avoidance contingency operated. Then, the right and left levers were inserted. Rats in all groups could press the right lever to obtain heroin infusions. The FR for heroin on the right lever was set to 1 for the first three sessions and increased across two-session blocks according to the sequence 2, 4, 8, 12, 18, 24, 32, 40, 52, 64, 80, 96, 128. For the No Alternative group, presses on the left lever had no programmed consequences. For the TOA Alternative group, TOA periods could be obtained by pressing the left lever. An FR-6 schedule for TOA reinforcers was used throughout the demand phase. The Heroin Alternative group could press the left lever for heroin infusions at a constant FR-6 price (i.e., regardless of the price of heroin on the right lever throughout the demand phase). This group was included as a positive control for substitution effects because heroin from one source should be a substitute for heroin from another source.

*Exp. 2: Effects of a concurrently available saccharin alternative on demand for heroin*

In Exp. 2, after learning to drink saccharin from the sipper tube in the operant chamber, all rats were first trained to press the left lever for 10-s insertions of the sipper tube of a bottle containing 0.2% saccharin solution. The FR was initially 1 and gradually increased over several sessions to 6. Then, all rats had surgery. After recovery from surgery, rats were assigned to either the Heroin With Saccharin group or the Heroin Only group. The Heroin With Saccharin group was trained on a procedure where the left and right levers were inserted simultaneously. Presses on the right lever were reinforced with a 0.03 mg/kg heroin infusion and presses on the left lever were reinforced by saccharin presentation. After 10 sessions with heroin available on an FR-1 schedule and saccharin available on an FR-6 schedule, the price of heroin was increased in two-session blocks according to the same sequence used in Exp. 1 plus the addition of two larger ratios, FR 192 and 256. The price of saccharin on the left lever remained at FR 6 throughout. The Heroin Only group was trained on the same procedure, except they had no saccharin alternative. Only the right lever was available to the Heroin Only group which they could press to self-administer heroin on the ascending series of FRs.

*Exp. 3: Effects of concurrently available heroin on demand for saccharin*

There were two groups in Exp. 3: the Saccharin With Heroin group and the Saccharin Only group. The Saccharin With Heroin group first had surgery and then was trained to press the right lever for heroin. Initially the FR was 1 and gradually increased to 6 over several sessions. Then, after first learning to drink saccharin from the sipper tube (2–4 sessions), the left lever was inserted simultaneously with the right lever. Presses on the left lever resulted in 10-s insertion of the saccharin sipper tube. For the first 10 sessions, the saccharin FR was 1 and the heroin FR was 6. Then the price of saccharin was increased over two-session blocks according to the same sequence used in Exp. 1. The price of heroin on the right lever was always FR 6. Rats in the Saccharin Only group were trained only with saccharin available for pressing the left lever. This group did not have surgery. After learning to drink from the saccharin sipper tube, this group was trained to press the left lever for 10-s saccharin reinforcers on an FR-1 schedule for 10 sessions before beginning the series of increasing FRs.

### Data analysis

Demand curves were fit to the heroin (Exp. 1 and 2) and saccharin (Exp. 3) consumption data using the zero-bounded demand model [15]. This model modifies the original Hursh and Silberberg [16] exponential demand model to accommodate zero consumption values. Formally, the zero-bounded demand model [15] is:

1 $${{IHS}}({{y}})={{IHS}}({{Q}_{0}})* {e}^{-\frac{\alpha }{{IHS}\left({Q}_{0}\right)}{Q}_{0}x}$$

As in the Hursh and Silberberg model, α represents the rate of change in the elasticity of demand and *Q*_0_ represents consumption at price 0. IHS represents the inverse hyperbolic sine transformation. The GraphPad Prism template available from the Institute for Behavior Resources (Baltimore, MD) website was used to fit the model to the data of the present experiments.

The primary demand curve metrics of interest were EV, which is 1/(α *100), and *Q*_0_. Both of these measures were provided as outputs of the Prism template mentioned above. Statistical analyses were performed on these measures derived from individual subjects’ demand curves. The model was also fit to the group mean data to produce the demand curves shown in figures. To evaluate whether a constant-price alternative reinforcer substituted for the reinforcer for which the price increased, log consumption of the constant-price reinforcer was plotted as a function of the log price of the increasing-price reinforcer and the slope of the linear regression line was obtained. The slopes for individual subjects’ data were used for statistical analysis. Using the threshold suggested by Bickel et al. [17] when using this method, a slope of 0.2 or greater was considered indicative of substitution.

The Type 1 error rate was set to 0.05 for all statistical tests. Group and Sex were between-subjects variables in 3 × 2 or 2 × 2 ANOVAs, depending on experiment. When there was a significant Group X Sex interaction, data were presented and analyzed separately for each sex. In Exp. 2, a significant interaction prompted exploratory 2 × 16 (Group X Price) repeated measures ANOVAs performed on the consumption data for each sex separately. Tukey posthoc tests or independent-sample *t*-tests were performed following significant ANOVA *F*-tests to further characterize group or sex differences. Although the experiments were not designed or powered for correlational analyses, posthoc exploratory Pearson partial correlations (controlling for sex) were performed to examine the relationship between *Q*_0_ and EV and between *Q*_0_ and the slope measure described above. These correlational analyses and discussion are presented in the Supplemental Results and Discussion.

## Results

*Exp. 1: Effects of a concurrently available TOA or heroin alternative on demand for heroin*

**Fig. 1:**
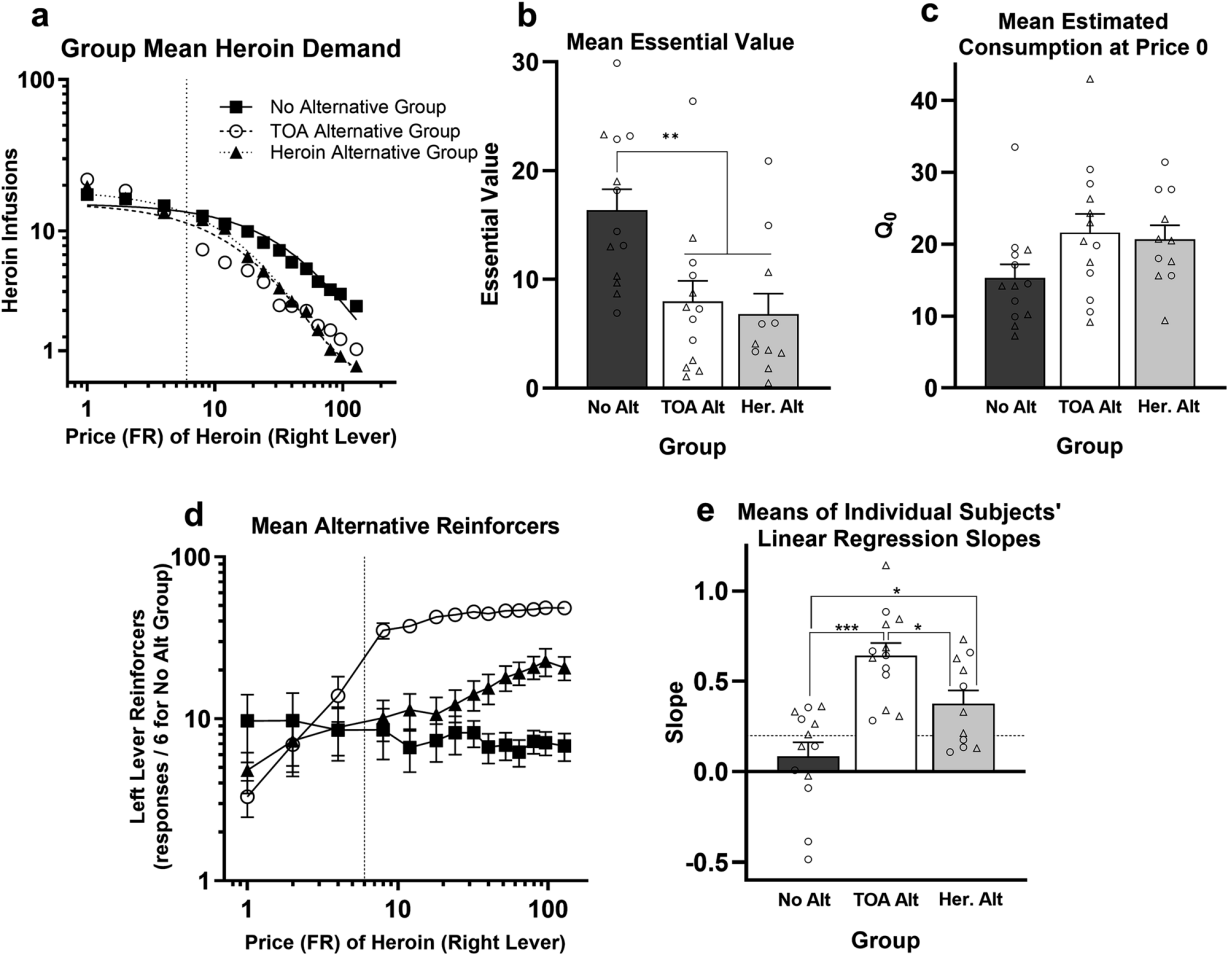
Experiment 1 results. **a** Demand curves fit to group mean consumption of heroin infusions (IHS transformed) across the prices (fixed ratios) for heroin on the right lever. The dotted vertical line represents the FR-6 price for TOA reinforcers or left-lever heroin infusions always available to rats in the TOA Alternative and Heroin Alternative groups, respectively. **b** Mean (±SEM) essential value for each of the three groups. Circles and triangles represent individual female and male rats, respectively. **p < 0.01. **c** Mean (±SEM) heroin *Q*_0_. **d** Mean (±SEM) consumption of left lever reinforcers as a function of the price of heroin on the right lever. The dotted vertical line represents the FR-6 price for left-lever reinforcers. For the No Alternative group, “reinforcers” where the number of left-lever responses divided by 6. **e** Mean (±SEM) of the slopes of the linear regression best fit lines relating log left-lever reinforcer consumption to log price of heroin on the right lever. The dashed horizontal line at 0.2 represents the threshold for identifying substitutes. *p < 0.05, ***p < 0.001.

Figure 1a shows group mean demand curves for the three groups. The No Alternative group (*n* = 13, 7 female / 6 male) worked harder than the TOA Alternative group (*n* = 13, 6 female/7 male) and the Heroin Alternative group (*n* = 11, 5 female/6 male) to defend heroin consumption when its price increased. Figure 1b shows that mean EV was significantly higher in the No Alternative group than in the TOA Alternative and Heroin Alternative groups, which did not differ from each other (main effect of Group, *F*[2,31] = 7.5, *p* = 0.002; Tukey posthocs, No Alternative group vs. TOA Alternative and Heroin Alternative group, both *p*s ≤ 0.006). The effect of group did not differ for females (circles in figures) vs. males (triangles; Group X Sex interaction, *F* < 1), but there was a significant main effect of Sex (*F*[1,31] = 6.7, *p* < 0.015), with female rats having generally higher heroin EV than males. The groups did not differ in heroin *Q*_0_ (Fig. 1c; *F*[2,31] = 2.8, *p* = 0.074). There was no main effect of Sex (*F*[1,31] = 1.1, *p* = 0.295) or Group X Sex interaction (*F*[2,31] = 1.7, *p* = 0.192) for heroin *Q*_0_.

Figure 1d shows that as the price of heroin on the right lever increased, so too did the number of TOA reinforcers and left-lever heroin infusions in the TOA Alternative and Heroin Alternative groups, respectively. In contrast, responding on the left lever in the No Alternative group did not change with price increases for heroin (Fig. 1d depicts “dummy reinforcers” for this group, i.e., the number of reinforcers that would have been obtained if an FR-6 schedule were in effect). Figure 1e shows the slope of the linear regressions relating log consumption of left-lever reinforcers to the log price of heroin on the right lever. A 2 × 2 (Group X Sex) ANOVA confirmed a significant effect of Group (*F*[2,31] = 15.1, *p* < 0.001), but no effect of Sex (*F*[1,31] = 3.1, *p* = 0.087) and no interaction (F < 1). Subsequent Tukey posthoc tests indicated that the mean slope in the No Alternative group was significantly lower than that in the TOA Alternative group (*p* < 0.001) and in the Heroin Alternative group (*p* = 0.022), with the latter two groups also differing from each other (*p* = 0.039). The lower bound of the 95% confidence intervals for both the TOA Alternative group (0.49, 0.79) and the Heroin Alternative group (0.22, 0.54) were above the 0.2 threshold for substitution suggested by Bickel et al. [17], whereas the interval included 0 for the No Alternative group (−0.08, 0.25).

*Exp. 2: Effects of concurrently available saccharin on heroin demand*

**Fig. 2:**
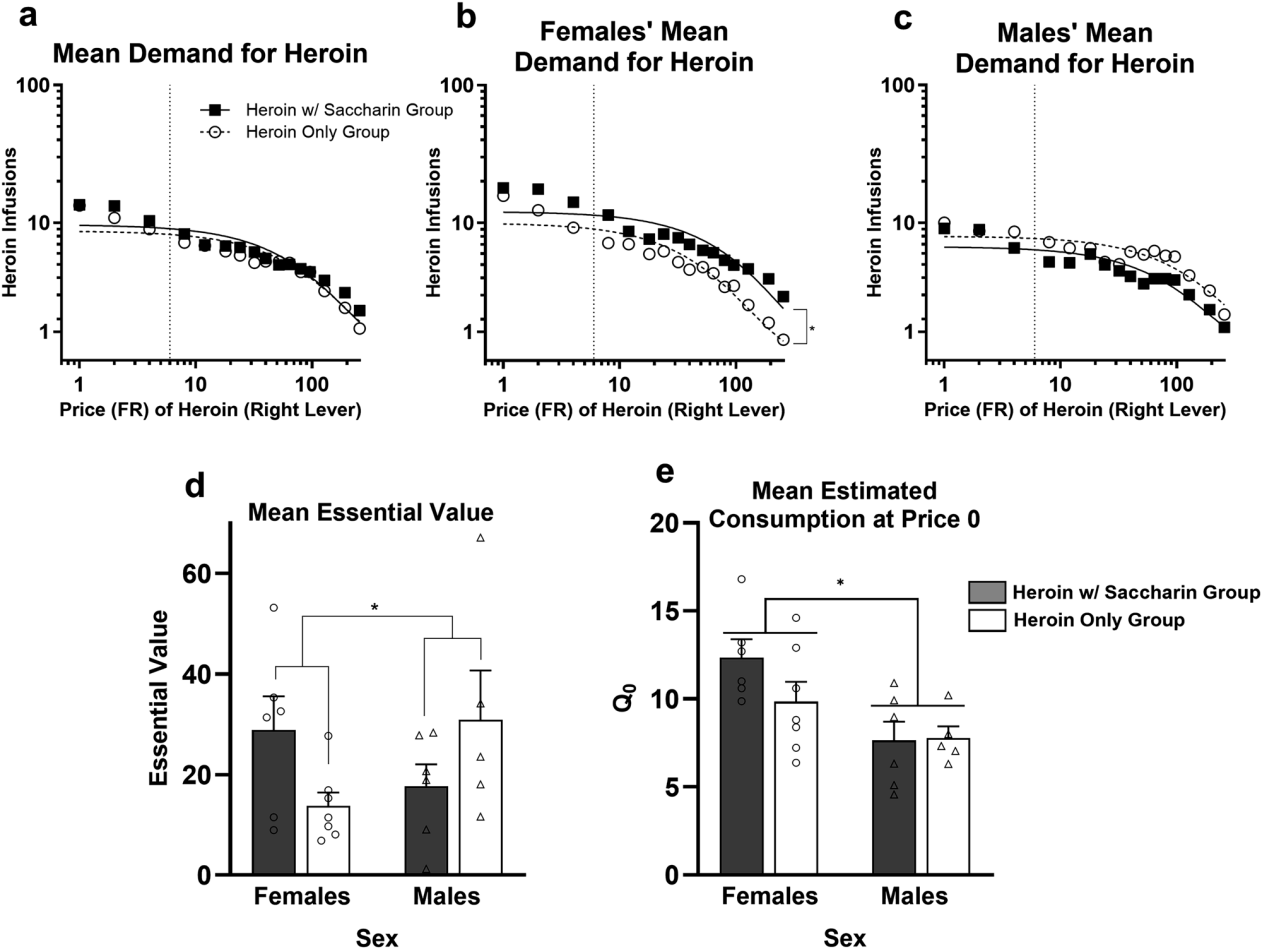
Experiment 2 results. **a** Demand curves fit to the combined-sexes group mean consumption of heroin infusions (IHS transformed) across the prices for heroin on the right lever. The dotted vertical line represents the FR-6 price for saccharin reinforcers always available to rats in the Heroin with Saccharin group. **b** Demand curves fit to group mean heroin consumption in female rats only. *p < 0.05, main effect of Group. **c** Demand curves fit to the group mean heroin consumption in male rats only. **d** Mean (±SEM) essential value for the female and male rats in each group. *p < 0.05, significant Group X Sex interaction. **e** Mean (±SEM) heroin *Q*_0_. *p < 0.05, for main effect of Group.

Figure 2a shows that when females and males are combined, group mean demand for heroin is highly similar for the Heroin With Saccharin (*n* = 12, 6 female, 6 male) and the Heroin Only (*n* = 12, 7 female, 5 male) groups. Figure 2b, c show separate results for females and males, respectively, because the ANOVA performed on EV (Fig. 2d) revealed a significant Group X Sex interaction (*F*[1,20] = 5.7, *p* = 0.027; no main effect of Group or Sex, both *F*s < 1). For females, EV tended to be higher in the Heroin With Saccharin group, whereas the opposite tendency was observed in the males. After correcting for multiple comparisons, independent-samples *t*-tests comparing groups separately within each sex were not significant (females, *t*(11) = 2.2, *p* = 0.050; males, *t*(9) = −1.3, *p* = 0.222). To further explore this interaction, 2 × 16 (Group X Price) repeated measures ANOVAs were performed on the number of heroin infusions obtained (Fig. 2b, c) for each sex separately. For the females, there was a significant main effect of Group (*F*[1,11] = 5.9, *p* = 0.033), with the Heroin With Saccharin group taking overall more heroin infusions during demand testing, and an effect of Price (*F*[15, 165] = 37.2, *p* < 0.001), but no interaction (*F* < 1). For the males, there was only an effect of Price (*F*[15, 135] = 20.3, *p* < 0.001) and no effect of Group (*F*[1,9] = 1.3, *p* = 0.279) or interaction (*F* < 1). Figure 2e shows that heroin *Q*_0_ was generally higher in females (*F*[1,20] = 10.5, *p* = 0.004). There was no effect of Group (*F*[1,20] = 1.3, *p* = 0.270) or interaction (*F*[1,20] = 1.6, *p* = 0.219) for heroin *Q*_0_.

**Fig. 3:**
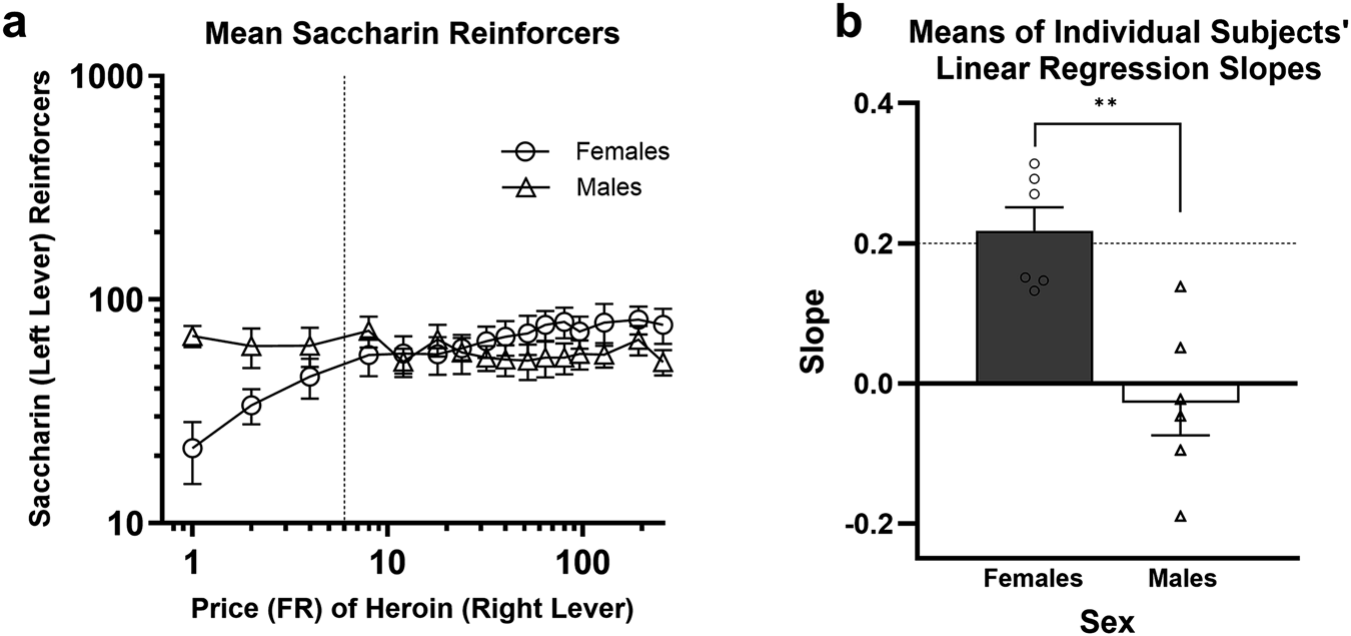
Experiment 2 results continued. **a** Mean (± SEM) consumption of saccharin reinforcers (IHS transformed) obtained by pressing the left lever as a function of the price of heroin on the right lever for female (circles) and male (triangles) rats in the Heroin with Saccharin group. The dotted vertical line represents the FR-6 price for saccharin reinforcers in the Heroin with Saccharin group. **b** Mean (±SEM) of the slopes of the linear regression best fit lines relating log saccharin consumption to log price of heroin on the right lever in the Heroin with Saccharin group. Circles and triangles represent individual female and male rats, respectively. The dashed horizontal line at 0.2 represents the threshold for substitution. **p < 0.01.

Figure 3a shows that, for the Heroin With Saccharin group, consumption of saccharin increased slightly with increases in the price of heroin for females, but was largely unchanged for males. An independent-samples *t*-test confirmed that the mean of the slopes (Fig. 3b) from linear regressions relating log saccharin consumption to log heroin price in females, 0.22 (95% CI: 0.13, 0.30), was significantly higher than the mean slope in males, −0.03 (95% CI: −0.15, 0.09) (*t*[10] = 4.3, *p* = 0.002).

*Exp. 3: Effect of concurrently available heroin on demand for saccharin*

**Fig. 4:**
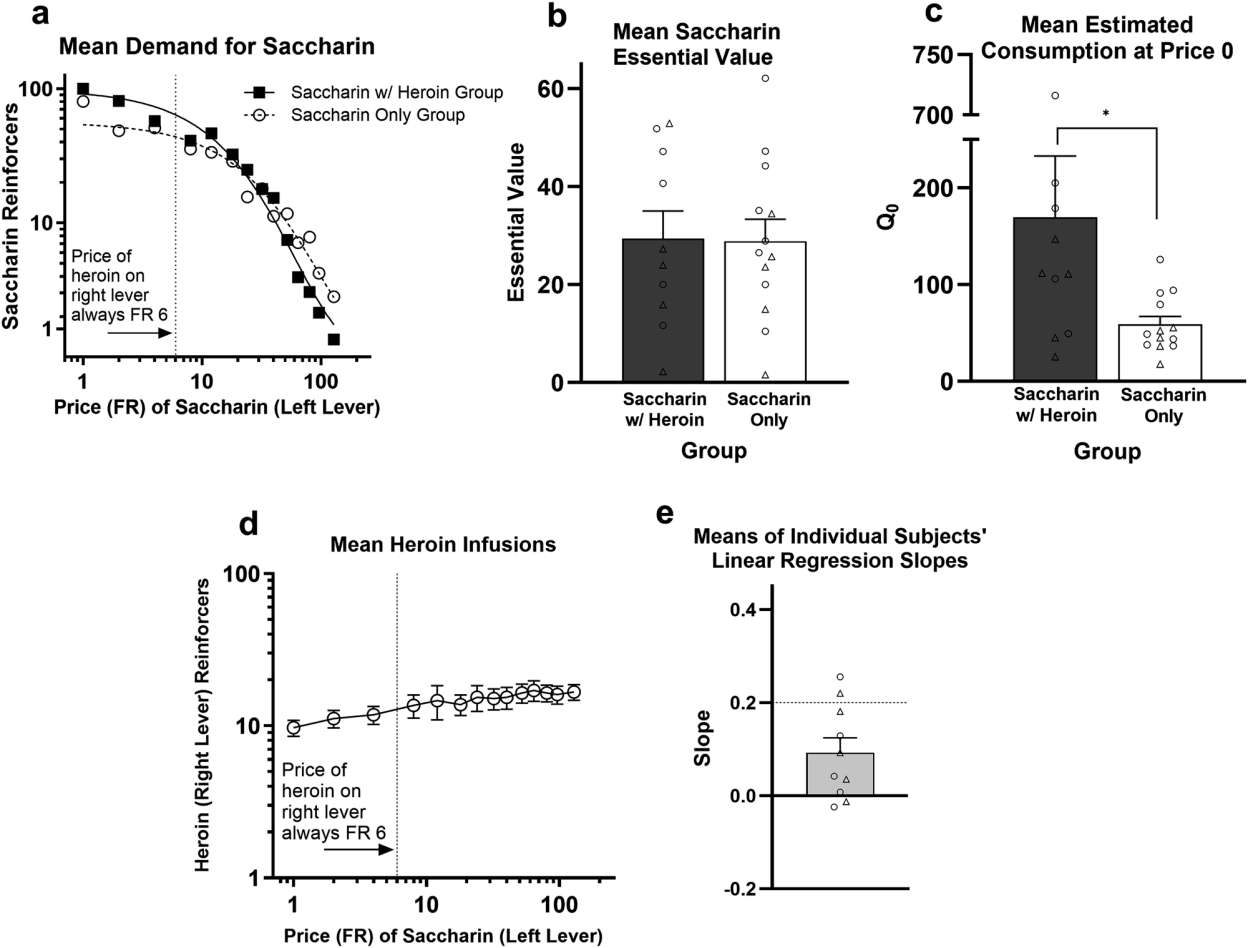
Experiment 3 results. **a** Demand curves fit to group mean consumption (IHS transformed) of saccharin reinforcers (left lever) across the prices (fixed ratios) of heroin on the right lever. **b** Mean (±SEM) essential value for the two groups. Circles and triangles represent individual female and male rats, respectively. **c** Mean (±SEM) heroin *Q*_0_. *p < 0.05. **d** Mean (±SEM) consumption of heroin infusions (right lever) as a function of the price of saccharin on the left lever in the Saccharin With Heroin group. The dotted line indicates that heroin was always available on the right lever on an FR-6 schedule. **e** Mean (±SEM) of the slopes of the linear regression best fit lines relating log heroin consumption to log price of saccharin in the Saccharin With Heroin group. The dashed horizontal line at 0.2 represents the threshold for substitution. *p < 0.05, ***p < 0.001.

The level of demand for saccharin in Exp. 3 (Fig. 4a) was generally higher than that observed for heroin in Exp. 1 and 2. Elasticity of demand for saccharin was similar in the Saccharin With Heroin group and the Saccharin Only group. For the EV measure (Fig. 4b), there was no effect of Group (*F* < 1), Sex (*F*[1,19] = 3.0, *p* = 0.102), or their interaction (*F* < 1). Saccharin *Q*_0_ data was log transformed to satisfy ANOVA assumptions of normality and heterogeneity of variance. The Saccharin With Heroin group had significantly higher saccharin *Q*_0_ than the Saccharin Only group (*F*[1,19] = 8.1, *p* = 0.010; Fig. 4c). Females had generally higher saccharin *Q*_0_ (main effect of Sex, *F*[1,19] = 5.5, *p* = 0.030), but there was no Group X Sex interaction (*F* < 1; Fig. 4c). While the Saccharin With Heroin and Saccharin Only groups earned means of 100 vs. 80 saccharin reinforcers, respectively, at FR 1, this difference was not significant (Group: *F*[1,19] = 2.0, *p* = 0.170; Sex (*F*[1,19] = 5.0, *p* = 0.038; Group X Sex interaction: *F* < 1). However, at FR 2, the mean difference (81 vs. 49) was significant (Group: *F*[1,19] = 5.3, *p* = 0.033; Sex: *F*[1,19] = 2.0, *p* = 0.169; Group X Sex interaction: *F* < 1). The general tendency at low prices for mean saccharin consumption in the Saccharin With Heroin to be higher (though not always significantly) than that of the Saccharin Only group likely contributed to the significant difference in saccharin *Q*_0_. Figure 4d shows that heroin consumption in the Saccharin With Heroin group was largely unaffected by the price of saccharin. The mean slope (Fig. 4e) was 0.09, with the upper bound of the 95% confidence interval (0.02, 0.16) below the 0.2 threshold for substitution. There was no sex difference in slopes (*t*[8] = −0.3, *p* = 0.756).

### Cross-experiment comparisons

Inspection of Figs. 1 and 2 suggests that potentially informative cross-experiment comparisons of heroin EV and heroin *Q*_0_ can be made. Specifically, it was noticed that heroin *Q*_0_ in the TOA Alternative and Heroin Alternative groups of Exp. 1 appeared higher than in the Heroin Only group of Exp. 2. Since there was no effect of group on heroin *Q*_0_ within either experiment, an exploratory 2 × 2 (Experiment X Sex) ANOVA was performed, which indicated that heroin *Q*_0_ was significantly higher in Exp. 1 than in Exp. 2 (*F*[1,57] = 32.9, *p* < 0.001) but there was no effect of Sex (*F*[1,57] = 2.4, *p* = 0.125) or Sex X Group interaction.

Additionally, it appeared that heroin EV may have differed between the groups in Exp. 1 and 2 that had no access to an alternative reinforcer, i.e., the No Alternative group in Exp. 1 and the Heroin Only group in Exp. 2. A 2 × 2 (Group X Sex) ANOVA performed on heroin EV from these two groups indicated that there was no main effects of Group (*F*[1,21] = 1.8, *p* = 0.200) or Sex (*F*[1,21] = 1.92, *p* = 0.180), but there was a significant Group X Sex interaction (*F*[1,21] = 5.4, *p* = 0.030). However, subsequent t-tests indicated that there was not a significant group difference for females (*t*[12] = 1.2, *p* = 0.263) or males (*t*[9] = −1.83, *p* = 0.100).

## Discussion

The main result of Exp. 1 was that the concurrent availability of TOA reduced the EV of heroin. This outcome is consistent with TOA being a substitute for heroin. Additional evidence of substitution comes from the observation that as the price of heroin increased (and heroin consumption declined), consumption of TOA reinforcers increased. The similarity of the results of the TOA Alternative group to those of the Heroin Alternative group, where the same heroin reinforcer was available on both levers and substitution should have occurred, provides additional support. The finding that TOA acted as a substitute for heroin within the demand approach used here is consistent with other recent studies using different methods to investigate choice between opioids and safety from shock [12, 18].

In Exp. 1, as the price of heroin on the right lever increased, the TOA group increased its consumption of TOA reinforcers to a significantly greater extent than the Heroin Alternative group increased its consumption of left-lever heroin infusions. This may appear to suggest that TOA was a more effective substitute for heroin than heroin itself. However, this outcome may have been due to rate-limiting effects of heroin preventing a larger increase in the Heroin Alternative group. If the infusions obtained by pressing the right-lever (Fig. 1a) are summed with the infusions obtained by pressing the left lever (Fig. 1d), it can be seen that the Heroin Alternative group took approximately 20 infusions in total per session throughout the experiment. The relative proportion of infusions obtained from pressing the right vs. left lever changed over the course of the experiment in the expected way as the relative prices across the levers changed. But, perhaps due to satiation or another rate-limiting effect of heroin, rats rarely took more than 20 infusions total. TOA reinforcer consumption may have increased to a greater degree than left-lever heroin infusions because potential rate-limiting factors did not limit TOA consumption in the same way.

The substitution relationship between TOA and heroin suggests that they serve a similar function. One possibility is heroin and TOA both reduce or prevent the experience of pain. However, it is unclear whether rats in Exp. 1 received sufficient heroin to produce antinociceptive effects. In previous experiments, the doses of i.v. heroin that produced antinociception were sometimes much higher than that used in Exp. 1. For example, in studies using the warm water tail withdrawal test, the (cumulative dose) ED_50_ for antinociceptive effects of i.v. heroin was found to be approximately 0.15–0.40 mg/kg [19, 20]. On the other hand, a bolus i.v. dose of 0.03 mg/kg heroin (same dose used in the present experiment) was found to produce antinociceptive effects as measured by paw withdrawal in response to mechanical stimulation, though this was observed in nerve-injured allodynic rats [21]. A bolus dose of 0.075 mg/kg i.v. heroin (equivalent to 2.5 infusions in the present experiment) produced antinociceptive effects as measured by paw withdrawal in response to thermal stimulation in rats [22]. The footshock used in Exp. 1 was different from the pain stimuli used in the studies described above, making it difficult to draw conclusions about antinociceptive effects from these cross-study comparisons.

Research examining the effects of experimenter-administered opioids on TOA-maintained behavior suggests that potential antinociceptive effects of opioids, or lack thereof, may be unrelated to the way in which opioids and TOA interact. Galizio and colleagues found that doses of intraperitoneal opioids (including morphine, methadone, and fentanyl) that increased, decreased, or had no effect on avoidance behavior consistently reduced numbers of TOA reinforcers obtained on variable-ratio or variable-interval schedules [23–25]. The finding that opioid doses that increased avoidance responding, and therefore presumably did not have antinociceptive effects, still reduced responding for TOA indicates that opioids’ effects on TOA do not depend on antinociception. Galizio et al. [25] proposed that rather than reducing nociception specifically, opioids may reduce the overall aversiveness of the avoidance situation and its associated cues, thereby reducing the reinforcing effectiveness of TOA periods. This hypothesis could potentially explain the substitution between TOA and heroin observed in Exp. 1. The reduction in overall aversiveness of the situation produced by either taking a heroin infusion or by earning a TOA reinforcer may have been functionally equivalent to some degree.

A limitation to conclusions drawn from the current study is that there were not separate groups of rats that were opioid-dependent. Previous experiments have found that long access to opioid self-administration, which produces signs of dependence, makes demand for opioids less elastic [26, 27]. It might therefore be expected that if rats here were opioid-dependent there would be a general increase in heroin EV. What is less certain is how opioid dependence would affect the way in which heroin interacted with the alternatives used here. If opioid dependence causes a change in the nature of heroin’s reinforcing effects (e.g., from positive reinforcement to negative reinforcement due to the removal of withdrawal symptoms), it might be expected that heroin would no longer interact with non-drug alternatives. However, Lenoir and Ahmed [26] found just the opposite – providing food (chow) as a concurrently available non-drug alternative on had a greater suppressive effect on demand for heroin, indicative of a substitution effect, in rats given long access to heroin than in rats given short access. This result suggests that opioid dependence can enhance economic interactions between heroin and some non-drug alternatives. Future research will be needed to determine how interactions between heroin and TOA or saccharin are affected by opioid dependence.

In Exp. 2 here, it was hypothesized that provision of a saccharin alternative, which a previous study found was a complement to heroin [12], would increase the EV of heroin. Collapsing across sex there was no effect of saccharin availability on heroin EV. However, there was a significant interaction whereby the effect of saccharin availability on heroin EV differed for females and males. Furthermore, in females, saccharin availability caused a general increase in heroin consumption (collapsed over prices), whereas it had no effect on heroin taking in males. These data provide some support for the notion that saccharin and heroin were complements in females. On the other hand, if heroin and saccharin were complements, it might have been expected that the decrease in consumption of heroin as its price increased in the Heroin With Saccharin group would be accompanied by decreases in the consumption of saccharin. Instead, increases in the price of heroin produced a small increase (but below the substitution threshold) in saccharin consumption in females and no change in males. In Exp. 3 here, concurrent availability of heroin had no impact on saccharin EV, but did increase saccharin *Q*_0_, a result consistent with Vandaele et al.’s [28] finding that heroin promotes saccharin taking and also with the notion that a complementary relation between heroin and saccharin exists. However, the decline in saccharin consumption caused by increases in its price was not accompanied by a decline in heroin consumption, as may be expected with complements. The somewhat mixed evidence for a complementary relation between heroin and saccharin in Exp. 2 and 3 here in comparison to the clear effect in Beasley et al. [12] could be due to procedural differences. Specifically, the income-compensated price change method used by Beasley et al. may be more sensitive in detecting complementary effects than the demand curve method used here.

Potential neurobiological mechanisms underlying a complementary relation between heroin and saccharin are the hedonic “hotspots” identified by Berridge and associates [29–31]. Microinjection of the mu-opioid receptor agonist DAMGO into a one cubic mm area of the nucleus accumbens shell caused a large increase in rats’ “liking” responses to the taste of a sweet sucrose solution and also produced increased consumption of chocolate candy [29]. Subsequent work found that microinjecting DAMGO into localized areas of cortex also produced an increase in liking reactions to a sweet taste [30], suggesting that there is a cortical-subcortical circuit that mediates the opioid-induced enhancement of liking of sweet tastes. Activation of this circuit by self-administered heroin, if it causes increased liking of saccharin, could potentially explain a complementary relation between heroin and saccharin. Learning that saccharin tastes better under the influence of heroin could potentially motivate rats to take more heroin when saccharin was concurrently available, as the female rats did in Exp. 2.

In comparing results across Exp. 1 and 2, it was noticed that heroin *Q*_0_ was generally higher in Exp. 1 than Exp. 2. It may have been that the aversive shock avoidance context in which Exp. 1 rats self-administered heroin caused the level of demand for heroin at low prices to be higher in Exp. 1 than in Exp. 2, where rats self-administered heroin in the absence of shock. If rats’ heroin taking were motivated in part by heroin’s analgesic or anxiolytic effects, negative reinforcement (removal of pain/stress) could have been an additional source of motivation for heroin in Exp. 1 that was absent in Exp. 2. This increased demand for heroin at low prices did not translate into reduced elasticity of demand as prices increased, however. When the No Alternative group of Exp. 1 was compared with the Heroin Only group of Exp. 2, there was no main effect of group on heroin EV. A potential explanation is that as the price of heroin increased and the No Alternative group of Exp. 1 received fewer heroin infusions, the analgesic/anxiolytic effects of heroin were reduced, leading rats to increase their rate of pressing the middle lever to avoid shock (see Supplementary Fig. S1a, where the No Alternative Group more than doubled their number of avoidance responses as the FR for heroin was increased from 1 to 32). The increased responding on the middle lever may have made it more difficult to complete the high ratios on the heroin lever. This increased response competition may contributed to the overall lack of a difference in heroin EV between the No Alternative group of Exp. 1 and the Heroin Only group of Exp. 2, which did not have to press the middle lever to avoid shock. These observations are made cautiously because they involve comparisons of groups from different experiments.

The finding in Exp. 1 that providing non-drug alternative ways of reducing stress or pain in the form of TOA can reduce opioid EV has potential clinical implications given the link between opioid EV and opioid use disorder. Some support for this idea comes from recent small-scale studies suggesting that non-pharmacological interventions for pain management, including ones based on cognitive behavioral therapy [32, 33], can reduce voluntary post-operative opioid use in humans (for review, see ref. [34]). The results of Exp. 2, on the other hand, show that, at least in female rats, availability of saccharin can cause an increase in the consumption of heroin. The substitutes for and complements to opioids in humans may differ from those in rats. Availability of sweet tastes may not be especially relevant for opioid use disorder (though see refs. [35, 36]), but there may be other non-drug alternatives that complement opioid use in humans. Future research will be needed to identify what they are.

Saccharin was chosen as the model of a heroin complement here because the goal was to test the hypothesis (described in the Introduction) that availability of a non-drug complement would increase heroin EV. To our knowledge, saccharin is the only non-drug alternative that has been shown to be complementary to opioids in rats [12]. In a previous study, potential substitutability/complementarity between social reinforcement and heroin was investigated in rats and they were found to be independent reinforcers [37]. Therefore, social interaction was not used here as the non-drug alternative because, as an independent with respect to heroin, it would not be expected to affect heroin EV. With independent goods, consumption of one is unaffected by consumption of the other [10].

The results of the current study show that the EV of an opioid depends on the broader behavioral economic context in which it is available. These findings are largely consistent with, but also suggest a caveat to, Acuff et al.’s contextualized reinforcer pathology model [38], which extends the original reinforcer pathology model [39], that emphasized individual differences in drug value and delay discounting, to include the important role of the larger context in which drug taking occurs. According to the contextualized reinforcer pathology model, the value of a drug is not constant, but rather depends on contextual variables, with the most important of them being the availability of non-drug alternatives. The results of Exp. 1 and 2 show that the value of heroin indeed depends on the availability of non-drug alternatives. However, whereas the contextualized reinforcer pathology model posits that alternative reinforcement is inversely related to substance use, the present results show that the impact of alternative reinforcers on drug demand depends not only on the availability of non-drug alternatives, but also on the way in which a particular non-drug alternative interacts with the drug. Increasing access to some non-drug alternatives may reduce demand for opioids (as TOA did in Exp. 1), but increasing access to other non-drug alternatives may have no effect on or even increase demand for opioids (as saccharin did in Exp. 2). Expanding the contextualized reinforcer pathology model to include the specific ways in which different non-drug alternatives interact with drugs may further our understanding of addiction.

## Supplementary information

Supplemental Methods, Result, and Discussion

## Data Availability

The Supplementary Materials contains much of the primary raw data for the three experiments. Additional data is available upon request to the first author.
