# Supplementary material for: Demand for heroin in rats: effects of non-drug alternative substitutes and complements
Source: Neuropsychopharmacology. 2025 May 9;50(12):1896–903. doi: 10.1038/s41386-025-02127-x (PMC12518876; doi:10.1038/s41386-025-02127-x)
Supplement: Supplementary file 1 — Supplemental Methods, Result, and Discussion [file 41386_2025_2127_MOESM1_ESM.docx]

**SUPPLEMENTAL METHODS**

**Subjects**

Female rats weighed approximately 200-260 g and male rats weighed approximately 280-515 g at the start of training. Rats were individually housed in Optirat Plus (Centennial, CO) home cages that were located in a temperature- and humidity-controlled vivarium room that had a 12-h light cycle, with lights on at 8:00 am. Experimental sessions were conducted during the light phase. Rats had free access to food and water in their home cages throughout experiments.

**Apparatus**

Each operant chamber measured 30.5 × 24 × 29 cm and had aluminum front and rear walls with clear polycarbonate side walls and a Med Associates grid floor. Three Med Associates retractable levers were located on the front wall of the chamber. A 100-mA cuelight was located above the left and right levers. A 100-mA houselight was located at the rear of the chamber near the ceiling. Heroin (0.1 mg/ml) dissolved in saline was infused at a rate of 3.19 ml/min by 10-ml syringes driven by Med Associates syringe pumps. Heroin was provided by the National Institute on Drug Abuse’s Drug Supply Program (Bethesda, MD). Tygon tubing extended from the 10-ml syringes to a 22-gauge rodent single-channel fluid swivel (Instech Laboratories, Plymouth Meeting, PA) and tether apparatus (In Exp. 1, P1 Technologies model C313CS/SPC, Roanoke, VA; in Exp. 2 & 3, Instech Laboratories model VABR1T/22) that descended through the ceiling of the chamber. Heroin was delivered to the subject through tubing that passed through the metal spring of the tether apparatus.

In Exp. 1, footshock was delivered through the grid floor of the chamber by Med Associates shocker/scrambler cards. A white noise stimulus (∼80 dB) that signaled when the avoidance contingency was in effect was generated by Med Associates ANL-926 cards and presented through a speaker located on the top left corner of the front wall of the chamber. In Exp. 2 and 3, saccharin reinforcers were provided by the operation of a Med Associates retractable sipper tube and bottle containing 0.2% saccharin dissolved in tap water. The aperture through which the sipper tube was inserted was located above the middle lever.

**Procedure**

***Experiment 1: Additional Procedural Details***

*Free-operant avoidance acquisition (middle lever).* Rats were first given two sessions of chamber habituation where they were placed in the chamber for 1-2 h with no levers or stimuli presented. On the next session, the shock intensity was set to 0 mA and increased in 0.1 mA increments until the rat showed an observable reaction. Rats were then shaped to press the middle lever to escape white noise and shock (each shock was preceded by 5 s of white noise) before being trained on the free-operant avoidance schedule. The shock intensity used was adjusted on an individual-subject basis so that the minimum intensity needed to maintain avoidance behavior was used. The mean shock intensities used during the final three avoidance acquisition sessions and for the rest of the experiment were 0.41, 0.39, and 0.42 mA for the No Alternative, TOA Alternative, and Heroin Alternative groups, respectively.

Avoidance sessions were signaled by insertion of the middle lever, presentation of the white noise, and illumination of the houselight. The avoidance schedule had a 35-s response shock (R-S) interval and a 5-s shock-shock (S-S) interval. This meant that each response postponed a shock by 35 s. If a rat did not respond for 35 s, the S-S interval began and the rat received a 0.5-s shock every 5 s until it made a response, which initiated the 35-s R-S interval. Each avoidance response also resulted in the white noise stimulus being turned off for 25 s. As rats acquired responding, the length of time that the white noise was turned off following a response was gradually decreased from 25 s to 0.5 s. Sessions lasted for 2 h or until 200 shocks were delivered. Rats were trained on the R-S 35-s, S-S 5-s avoidance schedule until successfully avoiding at least 65% of the maximum possible R-S shocks for three consecutive sessions.

*Surgery.* Following acquisition of free-operant avoidance, all rats had surgery to implant a jugular vein catheter, using procedures like those described by Beardsley and Shelton (2012) and Thomsen and Caine (2005). In Experiment 1, backmount catheter ports made by P1 Technologies (Roanoke, VA; model 313000BM-10/1) were used. In Experiments 2 and 3, backmount ports made by Instech Laboratories (Plymouth Meeting, PA; model VABR1B/22) were used. Approximately 3.5 cm of silicone or polyurethane tubing was inserted into the right jugular vein. (In a few instances, a rat was recatherized in the left vein if the original catheter became non-functional.) From this insertion site, an additional approximately 12 cm of silicone or polyurethane tubing passed under the skin to the midscapular region where it connected to the 22-gauge stainless steel tube of the backmount catheter port that was implanted subcutaneously. The spring tether in the chamber (P1 Technologies model C313CS/SPC in Exp. 1; Instech Laboratories model VABR1T/22 in Exp. 2 and 3) was attached to the top part of the backmount port that protruded through an opening in the skin. All surgery was conducted under ketamine (60 mg/kg) and xylazine (10 mg/kg) anesthesia. Rats were given two days of post-operative ketoprofen (5 mg/kg, s.c.). Rats were given 7 days to recover from surgery. Catheters were flushed after sessions with 0.1 ml of a saline solution containing 1.25 U/ml heparin and 0.08 mg/ml gentamicin. Catheter patency was tested at the end of the study and at occasional other times. A catheter was judged to be patent if ataxia was observed within 10 s of infusion of 0.02-0.03 ml of a solution containing 32 mg/ml ketamine and 3.8 mg/ml xylazine.

*Acquisition of right and left lever responding.* After at least a week of recovery from surgery, rats were assigned to one of the three groups. Assignment was made with the goal of matching groups in terms of shock intensity and mean avoidance response rate. All rats then learned to press the left and right levers in alternating sessions. The middle lever was inserted simultaneously with the left or right levers, and the avoidance schedule operated as described except for rats in the TOA Alternative group when a TOA reinforcer was earned. For all groups, during sessions when the right lever was inserted, presses on it were followed by a 0.03 mg/kg heroin infusion and illumination of the cue-light above the lever for 20-s.

The groups differed in terms of what happened during alternate sessions where the left lever was inserted. For the No Alternative group, the left lever was presented simultaneously with the middle lever, which rats could press on the avoidance schedule as usual. Presses on the left lever were recorded, but had no consequences for this group. For the TOA Alternative group, presses on the left lever resulted in a 120-s TOA period during which the avoidance schedule was suspended, the cue-light above the left lever was illuminated, the white noise and houselight were turned off, and all levers were retracted. For the Heroin Alternative group, presses on the left lever resulted in a 0.03 mg/kg heroin infusion and illumination of the cue-light above the left lever for 20 s. For the TOA Alternative and Heroin Alternative groups, the FR on the left lever was initially 1 and was gradually increased over sessions to FR-6, which was the left-lever FR for the remainder of the experiment. Rats had five sessions with each type of session, with approximately half of the rats in each group beginning the alternation with left-lever sessions and the other half beginning with right-lever sessions. Then all groups were trained on the demand procedure as described in the main text.

***Experiment 2: Additional Procedural Details***

*Saccharin bottle habituation.* All rats were first trained on a procedure designed to allow them to learn to drink from the saccharin bottle and to habituate them to the movement of the bottle as it was inserted and retracted. During sessions lasting 1 h, the bottle was inserted for 3-min periods, during which rats were free to drink from it. At the end of the 3 min, the bottle was retracted for a 10-s period before being re-inserted for another 3-min period. Rats were trained on this procedure for a minimum of two sessions. All rats learned to drink within 2-3 sessions.

*Acquisition of responding for saccharin (left lever).* All rats were then trained to press the left lever for saccharin reinforcers. Each lever press was followed by a 10-s insertion of the saccharin bottle along with 10-s illumination of the cue-light above the lever. The FR was initially 1, and was gradually increased over sessions to 6. For the Heroin with Saccharin group, the saccharin FR remained 6 for the rest of the experiment. For the Heroin Only group, this acquisition training was their last experience with saccharin and the left lever. Both groups were trained on this procedure so that they had equivalent experience with saccharin and with lever pressing before beginning heroin self-administration training. Following surgery and recovery from surgery, rats were assigned to groups and trained on the demand procedures as described in the main text. Rats were assigned to groups with the goal of matching them in terms of numbers on saccharin reinforcers obtained on the FR-6 schedule.

***Experiment 3: Additional Procedural Details***

*Heroin self-administration acquisition.* After surgery and recovery, rats in the Saccharin with Heroin group were trained to press the right lever for heroin. Each lever press resulted in a 0.03 mg/kg heroin infusion plus illumination of the cue-light above the lever for 20 s. Initially the FR was 1, and was gradually increased over sessions to 6, where it remained for the rest of the experiment for this group. The Saccharin Only group did not have surgery and did not experience the right lever or heroin.

*Saccharin Bottle Habituation.* Rats in both groups were trained on the same saccharin bottle habituation procedure used in Exp. 2. All rats learned to drink from the saccharin bottle within 2-4 sessions. Rats in both groups were then trained on the saccharin demand procedures described in the main text.

**SUPPLEMENTAL RESULTS AND DISCUSSION**

***Avoidance Behavior***

Fig. S1a shows mean avoidance responses (i.e., responses during the R-S 35-s interval) over the range of heroin prices tested (averaged over the two sessions at each price) in Exp. 1. Due to problems with ANOVA assumptions that could not be resolved by transforming the data, non-parametric statistics were used for avoidance data. At FR 1, when heroin consumption was relatively high in all groups (see Fig. 1a, main text), the groups made a comparable number of avoidance responses (no group difference, Kruskall-Wallis *H*[2] = 2.8, *p* = 0.249). As the price of heroin increased, and the number of TOA reinforcers earned by the TOA Alternative group increased (see Fig. 1d, main text), a group difference in number of avoidance responses emerged, with the TOA Alternative group making many fewer avoidance responses than the other two groups. A Kruskall-Wallis test performed on the avoidance responses made when the heroin FR was 128 at the end of the demand phase confirmed that there was a group difference (*H*[2] = 17.7, *p* < 0.001). Subsequent Mann-Whitney tests indicated that the TOA group made significantly fewer avoidance responses than either of the other two groups (*U*s ≤ 16.0, *p*s ≤ 0.002), which did not differ from each other (*U*[12,11] = 45.0, *p* = 0.211). Averaged over prices, there was no difference between females and males in the number of avoidance responses made (*U*[18,19] = 112, *p* = 0.075).

Fig. S1b shows the mean number of total escape responses per session. Escape responses included both those made during the 0.5-s shock and during the S-S 5-s interval. The pattern observed for escape responses largely parallels that observed for avoidance responses. When the FR for heroin on the right lever was 1, the groups made similar numbers of escape responses (no group difference, *H*[2] = 4.9, *p* = 0.085). When the price of heroin was FR 128, there was a group difference (*H*[2] = 19.9, *p* < 0.001). The TOA Alternative group made significantly fewer escape responses on the middle lever than the other two groups (both *U*s ≤ 7.0, both *p*s < 0.001), which did not differ from each other (*U*[12, 11] = 47, *p* = 0.260). Averaged across prices, there was no difference between female and male rats in number of escape responses (*U*[18,19] = 161.0, *p* = 0.775).

Fig. S1c shows the mean number of R-S shocks per session. These are the shocks received because rats let 35 s elapse without a response. For the No Alternative group and the Heroin Alternative group, the number of R-S shocks is equal to the number of escape responses, plus or minus 1 response (depending on whether the session ended in an R-S interval or an S-S interval). This is because an R-S shock marked the transition into the escape component (i.e., the S-S component) of the avoidance schedule and, once entering the escape component, a single response would return the rat to the avoidance component (i.e., the R-S component) of the avoidance schedule. For the TOA alternative group, the number of R-S shocks varied slightly from the number of escape responses because this group could also escape from the S-S component by earning a TOA reinforcer. Statistical analyses confirmed that at FR 1 for heroin there was no significant group effect (*H*[2] = 4.2, *p* = 0.124). At FR 128, there was a group effect (*H*[2] = 18.5, *p* < 0.001), with the TOA Alternative group receiving significantly fewer R-S shocks than the other two groups (both *U*s ≤ 9.5, both *p*s < 0.001), which did not differ from each other (*U*[12, 11] = 47, *p* = 0.260). Averaged across prices, there was no difference between female and male rats in number of R-S shocks (*U*[18,19] = 163.0, *p* = 0.822).

Fig. S1d shows the numbers of S-S shocks received per session in each group at each of the heroin prices on the right lever. At FR 1 for heroin, the groups received a comparable number of shocks (*H*[2] = 2.0, *p* = 0.365). This result is somewhat surprising because the TOA group could earn 2-min signaled reprieves from the avoidance schedule by completing the FR-6 requirement on the left lever. However, as Fig. 1d of the main text shows, they only obtained a mean of about 3 TOA reinforcers per session when the price of heroin was FR 1. Only when the FR for heroin on the right lever surpassed the FR for TOA on the left lever (i.e., at heroin FR 8) did rats take a large number of TOA reinforcers, causing the number of S-S shocks received by the TOA Alternative group to begin to diverge from the other two groups. As heroin price increased to FR 128, group differences emerged (*H*[2] = 19.3, *p* < 0.001; subsequent Mann-Whitney U tests revealed that each group differed from every other group, all *U*s ≤ 30.5, all *p*s ≤ 0.027). The TOA group received the fewest S-S shocks when the price of heroin was high, which is not surprising because they earned many TOA periods during those sessions. The Heroin Alternative group received more S-S shocks than the No Alternative group. Because there was no difference between these groups in the numbers of escape responses (Fig. S1b), this meant that Heroin Alternative group rats were waiting longer in the S-S component, and receiving a shock every 5 s, before making an escape response than rats in the No Alternative group.

***Correlations Between* Q*_0_ and EV and Between* Q*_0_ and Slope***

Fig. S2 illustrates for each group in Exp. 1 the relationship between heroin *Q*_0_ and heroin EV (top panels) and the relationship between heroin *Q*_0_ and the slope of the best fit line describing the change in consumption of the left-lever alternative as a function of the price of heroin on the right lever. Circles represent female rats and triangles represent males. For the TOA Alternative group, there was a negative correlation (controlling for sex with partial Pearson *r*) between heroin *Q*_0_ and heroin EV (*r*[10] = -0.58, *p* = 0.047). These correlations for the No Alternative group (*r*[10] = 0.31, *p* = 0.32) and the Heroin Alternative Group (*r*[8] = 0.59, *p* = 0.073) did not reach significance. There was no significant correlation between heroin *Q*_0_ and the slope measure for any group (*r*s between -0.32 and 0.23, all *p*s > 0.3).

Fig. S3 illustrates for both groups in Exp. 2 the relationship between heroin *Q*_0_ and heroin EV (top panels) and the relationship between *Q*_0_ and slope in the Heroin with Saccharin group (bottom panel). There was no significant correlation between *Q*_0_ and EV in either group (*r*s between 0.12 and 0.43, both *p*s > 0.7). There was no significant correlation between *Q*_0_ and slope for the Heroin with Saccharin group (*r*[9] = 0.08, *p* = 0.820).

Fig. S4 shows for both groups in Exp. 3 the relationship between log saccharin *Q*_0_ and saccharin EV (top panels) and the relationship between log saccharin *Q*_0_ and slope for the Saccharin with Heroin group. (Log saccharin *Q*_0_ was used in Exp. 3 due to normality concerns.) There was a significant positive correlation between saccharin *Q*_0_ and EV in the Saccharin Only group (*r*[10] = 0.76, *p* = 0.005), but not the Saccharin with Heroin group (*r*[7] = 0.13, *p* = 0.740). There was a significant positive correlation between log saccharin *Q*_0_ and the slope measure in the Saccharin with Heroin group (*r*[7] = 0.74, *p* = 0.022).

Because this study was not designed or powered for correlational tests, conclusions drawn from this posthoc exploratory analysis must be made with caution. In Exp. 1, the finding of a marginally significant negative correlation between heroin *Q*_0_ and EV in the TOA group indicates that rats that had higher heroin intake at low prices tended to be less persistent in defending heroin intake as its price increased. Why this was the case is unclear. It might have been expected that rats with *lower* heroin intake at low prices would more quickly decrease heroin consumption as price of heroin increased and TOA became a more attractive alternative. Interestingly, though not significant, in the Heroin Alternative group the rats with higher heroin intake at low prices tended to be more persistent in defending intake of heroin obtained from pressing the right lever as its price increased. This opposite tendency observed across the TOA Alternative and Heroin Alternative groups with respect to the relationship between heroin *Q*_0_ and EV might suggest that the way in which the left lever alternative (TOA or heroin, respectively) substituted for heroin on the right lever may have differed. On the other hand, in both groups, there was a similarly small positive (non-significant) correlation between *Q*_0_ and the slope reflecting the degree to which left-lever reinforcer consumption increased as the price of heroin on the right lever increased. This indicates that the degree to which left lever reinforcer consumption increased mostly did not depend on right-lever heroin consumption at low prices in both groups. Future research will be needed to unravel these potentially complex interrelationships among *Q*_0_, EV, and substitutability.

In Exp. 2, there were no significant correlations. However, it is worth noting that the correlation between heroin *Q*_0_ and heroin EV in the Heroin Only group (*r* = 0.43) was comparable to that observed in the No Alternative group of Exp. 1 (*r* = 0.31). In both groups, heroin was the only reinforcer available. Finding a similar moderate correlation (non-significant) in both groups provides some evidence of cross-experiment replicability and suggests that heroin intake at low price is only a moderate predictor of how hard rats will work to defend heroin intake when it is the only reinforcer available.

In Exp. 3, for the Saccharin Only group, there was a strong positive correlation (*r* = 0.76) between saccharin *Q*_0_ and saccharin EV. This suggests that when saccharin is the only reinforcer available, intake at low price is a good predictor of how hard rats will work to defend saccharin intake as its price increases. However, when heroin was concurrently available, as in the Saccharin with Heroin group, saccharin intake at low prices was no longer a good predictor of rats’ willingness to defend saccharin intake as its price increased (*r* = 0.13). Additionally, in the Saccharin with Heroin group those rats with the highest saccharin intake at low prices tended to show the largest increase in heroin taking when the price of saccharin increased (*r* = 0.74 for correlation between saccharin *Q*_0_ and slope). This was the only group out of five across the three experiments that showed a significant relationship between *Q*_0_ and slope (all other *r*s ≤ 0.23), and this was the only group of those five that worked for saccharin rather than heroin, potentially suggesting that saccharin *Q*_0_ is a more informative predictor of subsequent behavior than heroin *Q*_0_. Why this should be the case is unclear. Future research that is powered for correlational research will be needed to better investigate the potential relationships described above.

| **Table S1. Numbers of heroin infusions at each FR for individual subjects in Experiment 1** | | | | | | | | | | | | | | |
| --- | --- | --- | --- | --- | --- | --- | --- | --- | --- | --- | --- | --- | --- | --- |
|  | | | | | | | | | | | | | | |
| No Alternative Group | | | | |  |  |  |  |  |  |  |  |  |  |
| Subject | FR1 | FR2 | FR4 | FR8 | FR12 | FR18 | FR24 | FR32 | FR40 | FR52 | FR64 | FR80 | FR96 | FR128 |
| f2 | 25 | 12.5 | 14.5 | 11 | 17 | 12 | 6.5 | 17.5 | 6 | 8 | 6 | 4 | 3.5 | 2.5 |
| f6 | 20.5 | 22 | 21 | 18.5 | 14 | 12.5 | 12.5 | 9 | 8.5 | 7 | 6.5 | 5.5 | 4.5 | 5 |
| f18 | 29 | 35.5 | 30 | 31.5 | 27 | 21 | 15 | 10 | 8 | 7.5 | 6 | 5.5 | 4.5 | 3.5 |
| m19 | 15 | 14 | 6.5 | 5.5 | 5 | 7 | 6.5 | 5.5 | 6 | 5 | 4.5 | 4 | 4 | 3.5 |
| m21 | 16 | 16 | 15 | 9.5 | 9 | 8 | 8 | 6 | 5.5 | 5.5 | 4.5 | 3.5 | -- | -- |
| m23* | 18.5 | 8.5 | 5 | 1.5 | 0 | 0 | 0.5 | 0.5 | 0 | 0 | 0 | 0 | 0 | 0 |
| m1 | 10 | 8.5 | 7 | 4 | 6.5 | 3.5 | 5.5 | 4.5 | 3 | 2.5 | 2 | 2.5 | 2 | 2 |
| f33 | 13 | 14.5 | 17.5 | 16 | 12 | 15.5 | 12.5 | 6.5 | 7 | 7.5 | 6.5 | 7 | 6.5 | 6 |
| m38 | 16.5 | 17 | 15 | 15 | 10 | 11.5 | 10 | 5 | 5 | 3 | 3 | 2 | 2 | 0 |
| f43 | 21 | 18 | 17.5 | 12 | 13 | 10.5 | 8.5 | 8.5 | 9 | 8 | 2.5 | 2 | 3.5 | 2.5 |
| f45 | 10.5 | 15.5 | 10.5 | 10 | 6 | 5 | 5 | 4 | 4.5 | 2 | 2 | 2 | 1 | 1 |
| m34 | 16.5 | 10.5 | 13 | 6.5 | 7 | 5 | 4.5 | 4.5 | 4 | 3.5 | 3 | 3 | 3 | 2 |
| m48 | 18.5 | 16.5 | 14 | 13 | 11 | 8.5 | 7 | 9 | 8 | 8 | 7.5 | 5.5 | 5.5 | 3.5 |
| f93 | 14 | 10.5 | 8.5 | 9.5 | 6.5 | 8 | 6 | 5 | 4.5 | 3.5 | 3.5 | 3.5 | 3 | 3 |
| Median | 16.5 | 15 | 14.25 | 10.5 | 9.5 | 8.25 | 6.75 | 5.75 | 5.75 | 5.25 | 4 | 3.5 | 3.5 | 2.5 |

|  | | | | | | | | | | | | | | |
| --- | --- | --- | --- | --- | --- | --- | --- | --- | --- | --- | --- | --- | --- | --- |
| TOA Alternative Group | | | | |  |  |  |  |  |  |  |  |  |  |
| Subject | FR1 | FR2 | FR4 | FR8 | FR12 | FR18 | FR24 | FR32 | FR40 | FR52 | FR64 | FR80 | FR96 | FR128 |
| m9 | 16.5 | 11 | 10 | 3.5 | 2.5 | 0.5 | 0 | 0 | 0 | 0 | 0 | 0 | 0 | 0 |
| m13 | 14 | 11.5 | 8 | 6 | 5 | 4.5 | 4.5 | 4.5 | 4 | 4 | 3 | 2.5 | 2 | 1 |
| m11 | 10.5 | 10.5 | 10.5 | 1 | 0.5 | 0.5 | 0 | 0 | 0 | 0 | 0 | 0 | 0 | 0 |
| f12 | 27.5 | 29 | 13.5 | 3 | 9.5 | 9 | 7 | 7.5 | 7 | 6 | 5.5 | 6 | 7 | 5.5 |
| m7 | 19.5 | 16.5 | 15 | 4 | 0.5 | 0 | 0 | 0 | 0 | 0 | 0 | 0 | 0 | 0 |
| f16 | 22 | 18 | 10.5 | 11.5 | 6.5 | 6.5 | 7 | 4 | 1.5 | 2.5 | 3 | 2.5 | 1 | 1 |
| f26 | 29.5 | 30.5 | 22 | 8 | 6 | 7 | 6 | 3 | 4.5 | 1 | 1 | 0.5 | 0 | 0 |
| m36 | 27 | 21 | 13 | 10 | 8 | 9.5 | 7.5 | 2 | 4 | 4 | -- | -- | -- | -- |
| f37 | 22.5 | 13 | 12 | 7 | 6 | 5.5 | 4.5 | 4 | 3 | 3 | 2.5 | 3.5 | 3.5 | 3 |
| f39 | 28 | 19.5 | 17 | 14.5 | 10 | 8 | 6.5 | 6 | 6 | 7 | -- | -- | -- | -- |
| m42 | 22 | 18.5 | 15.5 | 12.5 | 8.5 | 10.5 | 9 | 6.5 | 6.5 | 6 | 6.5 | 4.5 | 2 | 1 |
| m94^†#^ | 16 | 21.5 | 29 | 37.5 | 37.5 | 34 | 32.5 | 28.5 | 29.5 | 25.5 | 29 | 31 | 25.5 | 24 |
| f95 | 29.5 | 20.5 | 15.5 | 7.5 | 11.5 | 3.5 | 5 | 0.5 | 1 | 1 | 0 | 0 | 0 | 0 |
| m98 | 15.5 | 20.5 | 10.5 | 8 | 4 | 4 | 0 | 0 | 0 | 0 | 0 | 0 | 0 | 0 |
| Median | 22 | 19.5 | 13.5 | 8 | 6.5 | 6.5 | 6 | 4 | 4 | 3 | 2.5 | 2.5 | 1 | 1 |

| **Table S1 cont’d.** | | | | | | | | | | | | | | |
| --- | --- | --- | --- | --- | --- | --- | --- | --- | --- | --- | --- | --- | --- | --- |
|  | | | | | | | | | | | | | | |
| Heroin Alternative Group | | | | |  |  |  |  |  |  |  |  |  |  |
| Subject | FR1 | FR2 | FR4 | FR8 | FR12 | FR18 | FR24 | FR32 | FR40 | FR52 | FR64 | FR80 | FR96 | FR128 |
| m3 | 12.5 | 11 | 8.5 | 7.5 | 2 | 1.5 | 0.5 | 0.5 | 0 | 0.5 | 0 | 0 | 0 | 0 |
| f22 | 42.5 | 23.5 | 21 | 21.5 | 18 | 18 | 14.5 | 10.5 | 9.5 | 4.5 | 7 | 5 | 4.5 | 3 |
| f8^#*^ | 6 | 12.5 | 9.5 | 8 | 6 | 2.5 | 1 | 0.5 | 0 | 0 | 0 | 0 | 0 | 0.5 |
| f14 | 25.5 | 14.5 | 12.5 | 10.5 | 5.5 | 4.5 | 2 | 0.5 | 0 | 0 | 0 | 0 | 0 | 0 |
| m17 | 13.5 | 13.5 | 14 | 16.5 | 16.5 | 14 | 14 | 14.5 | 16 | 6 | 0.5 | 0 | 0 | 0 |
| m25 | 16.5 | 13 | 13 | 10 | 6 | 3.5 | 1.5 | 1 | 1 | 0.5 | 0.5 | 0 | 0 | 0 |
| f4 | 15 | 15.5 | 15.5 | 8 | 8 | 5.5 | 5.5 | 2.5 | 2.5 | 2 | 2 | 1 | 1 | 1 |
| f35 | 19 | 16.5 | 14 | 14.5 | 7 | 6 | 2 | 4 | 3 | 2 | 1 | 1.5 | 1.5 | 0.5 |
| m46 | 7 | 4.5 | 1.5 | 1 | 1 | 0.5 | 0.5 | 0 | 0 | 0 | 0 | 0 | 0 | 0 |
| m40 | 11.5 | 9.5 | 10 | 9 | 10.5 | 6.5 | 5.5 | 3.5 | 0.5 | 0 | 0 | 0 | 0 | 0 |
| m32 | 14.5 | 14 | 10 | 11.5 | 10 | 6 | 2 | 0.5 | 0.5 | 0 | 0.5 | 0 | 0 | 0 |
| f31 | 38.5 | 45 | 24.5 | 19.5 | 29 | 6.5 | 9.5 | 6 | 1.5 | 13 | 8 | 4 | 2 | 1 |
| m92^*^ | 24.5 | 21.5 | 19.5 | 11 | 19 | 5.5 | 4 | 3 | 1 | 0 | 0 | 0.5 | 0 | 0 |
| Median | 15 | 14 | 13 | 10.5 | 8 | 5.5 | 2 | 2.5 | 1 | 0.5 | 0.5 | 0 | 0 | 0 |

Note: The following notes apply to this and subsequent tables. Numbers of reinforcers are averaged over the two sessions at each FR. “--“ indicates that a rat did not complete an FR (due to catheter problems or illness). *, †, or # symbols indicate that a rat met Stein et al.’s (2015) reversal from zero, trend, or bounce criteria, respectively, for non-systematic demand data. Subject identifiers starting with “f” were female rats and those starting with “m” were male rats.

| **Table S2. Numbers of heroin infusions at each FR for individual subjects in Experiment 2** | | | | | | | | | | | | | | | | | | | | | | | | | | | |  |
| --- | --- | --- | --- | --- | --- | --- | --- | --- | --- | --- | --- | --- | --- | --- | --- | --- | --- | --- | --- | --- | --- | --- | --- | --- | --- | --- | --- | --- |
|  | | | | | | | | | | | | | | | | | | | | | | | | | | | |  |
| Heroin With Saccharin Group | | | | | | |  | | |  | |  | |  | |  | |  | |  | |  | | |  |  | |  |
| Subject | FR1 | FR2 | FR4 | FR8 | FR12 | FR18 | | FR24 | FR32 | | FR40 | | FR52 | | FR64 | | FR80 | | FR96 | | FR128 | | FR192 | FR256 | | |  |  |
| f51^#^ | 8 | 12.5 | 21.5 | 14 | 3 | 3.5 | | 3.5 | 1 | | 2 | | 3.5 | | 3.5 | | 3.5 | | 2.5 | | 2.5 | | 2 | -- | | |  |  |
| m56 | 11.5 | 13 | 5.5 | 7.5 | 6 | 10 | | 10.5 | 9 | | 9 | | 6.5 | | 7.5 | | 5.5 | | 4.5 | | 4 | | 2 | 2.5 | | |  |  |
| m62 | 9.5 | 5.5 | 6 | 4 | 3 | 1 | | 0 | 0 | | 0 | | 0 | | 0 | | 0 | | 0 | | 0 | | 0 | 0 | | |  |  |
| m60 | 7.5 | 5.5 | 5 | 3.5 | 3.5 | 4 | | 2.5 | 2 | | 2 | | 2 | | 2 | | 2 | | 2.5 | | 2 | | 1.5 | 0 | | |  |  |
| f59b | 27.5 | 22 | 19 | 12.5 | 11.5 | 12.5 | | 13 | 11.5 | | 8 | | 6 | | 7.5 | | 7 | | 7 | | 7 | | 3.5 | 2.5 | | |  |  |
| f55^#^ | 11.5 | 20.5 | 21.5 | 12.5 | 13.5 | 10.5 | | 10 | 9 | | 10 | | 10 | | 10.5 | | 9.5 | | 7 | | 7.5 | | 5 | 4.5 | | |  |  |
| m72 | 10 | 14.5 | 10 | 6.5 | 6.5 | 8 | | 6 | 5.5 | | 5 | | 5 | | 7 | | 7 | | 7 | | 4.5 | | 3 | 1.5 | | |  |  |
| f73 | 21.5 | 19.5 | 13.5 | 11 | 7.5 | 7 | | 11.5 | 12.5 | | 12.5 | | 10.5 | | 9 | | 7.5 | | 7 | | 6.5 | | 6 | 4.5 | | |  |  |
| f75 | 14 | 19.5 | 17 | 10.5 | 7.5 | 3.5 | | 3 | 2 | | 2.5 | | 2.5 | | 3 | | 3 | | 2.5 | | 2.5 | | 1.5 | 2 | | |  |  |
| f77 | 10 | 12 | 8.5 | 12 | 10.5 | 10.5 | | 8.5 | 9.5 | | 7.5 | | 6.5 | | 7.5 | | 5.5 | | 6 | | 3.5 | | 4 | 3.5 | | |  |  |
| m80 | 8 | 6.5 | 5 | 3 | 4.5 | 3 | | 3 | 5 | | 4.5 | | 4 | | 3 | | 4.5 | | 3.5 | | 3 | | 2 | 1 | | |  |  |
| m82 | 7.5 | 8 | 7 | 5.5 | 6 | 8.5 | | 6.5 | 4 | | 2.5 | | 2.5 | | 2.5 | | 3 | | 4 | | 2.5 | | 3 | 2 | | |  |  |
| fg83 | 20 | 17.5 | 12.5 | 9 | 7 | 6.5 | | 6.5 | 6.5 | | 5.5 | | 6.5 | | 4.5 | | 4.5 | | 3.5 | | 4.5 | | 6.5 | 3 | | |  |  |
| fg84 | 14.5 | 15 | 14 | 13 | 7.5 | 5 | | 6.5 | 4 | | 5 | | 5 | | 4 | | 3.5 | | 2.5 | | 2.5 | | 0.5 | 0 | | |  |  |
| Median | 10.75 | 13.75 | 11.25 | 9.75 | 6.75 | 6.75 | | 6.5 | 5.25 | | 5 | | 5 | | 4.25 | | 4.5 | | 3.75 | | 3.25 | | 2.5 | 2 | | |  |  |

|  | | | | | | | | | | | | | | | | | | | | | | | | | | | |  |
| --- | --- | --- | --- | --- | --- | --- | --- | --- | --- | --- | --- | --- | --- | --- | --- | --- | --- | --- | --- | --- | --- | --- | --- | --- | --- | --- | --- | --- |
| Heroin Only Group | | | | | | |  | | |  | |  | |  | |  | |  | |  | |  | | |  |  | |  |
| Subject | FR1 | FR2 | FR4 | FR8 | FR12 | FR18 | | FR24 | FR32 | | FR40 | | FR52 | | FR64 | | FR80 | | FR96 | | FR128 | | FR192 | FR256 | | |  |  |
| m52 | 8 | 8 | 7 | 4 | 4 | 3.5 | | 3 | 4 | | 5 | | 4.5 | | 5 | | 3 | | 2.5 | | 1.5 | | 1 | 0 | | |  |  |
| m54 | 14 | 11.5 | 9 | 7 | 5.5 | 5 | | 3 | 3 | | 4 | | 4 | | 4 | | 4 | | 3.5 | | 3.5 | | 2.5 | 1.5 | | |  |  |
| f57 | 24.5 | 26.5 | 15 | 5 | 5.5 | 2.5 | | 6 | 5 | | 4 | | 8 | | 3 | | 2 | | 1 | | 0.5 | | 0 | 0 | | |  |  |
| f61 | 9 | 8 | 7.5 | 6.5 | 4.5 | 5.5 | | 5 | 4 | | 3 | | 3 | | 2 | | 2 | | 1.5 | | 0.5 | | 0 | 0 | | |  |  |
| f71 | 15 | 9 | 6.5 | 7 | 6.5 | 7 | | 7.5 | 6.5 | | 4.5 | | 3.5 | | 3.5 | | 3.5 | | 3.5 | | 2.5 | | 2 | 1 | | |  |  |
| m78 | 11.5 | 9.5 | 8.5 | 6.5 | 6 | 5.5 | | 4 | 4.5 | | 5.5 | | 4.5 | | 5.5 | | 5 | | 4.5 | | 4 | | 3.5 | 3 | | |  |  |
| f79 | 16 | 14 | 13 | 12.5 | 12 | 9.5 | | 11 | 6 | | 6.5 | | 7.5 | | 8.5 | | 5 | | 6 | | 5 | | 3 | 2 | | |  |  |
| f81 | 9 | 6.5 | 5.5 | 5 | 6 | 4.5 | | 4.5 | 3 | | 3.5 | | 3.5 | | 3.5 | | 3 | | 2.5 | | 2 | | 1.5 | 0 | | |  |  |
| m74b^#^ | 9.5 | 11 | 14.5 | 13 | 14.5 | 14.5 | | 14 | 13 | | 15 | | 17 | | 16.5 | | 14.5 | | 16.5 | | 11.5 | | 9.5 | 8 | | |  |  |
| m58b | 9.5 | 7.5 | 11.5 | 11.5 | 10.5 | 11 | | 8.5 | 7 | | 8.5 | | 8.5 | | 9 | | 9 | | 9 | | 7 | | 5.5 | 4 | | |  |  |
| fg81 | 22 | 12.5 | 7 | 4 | 4 | 3.5 | | 2.5 | 3 | | 3 | | 2.5 | | 3.5 | | 2.5 | | 2.5 | | 2 | | 1.5 | 1 | | |  |  |
| fg82 | 14.5 | 9.5 | 9.5 | 9 | 9.5 | 8 | | 6 | 7.5 | | 6 | | 4 | | 4.5 | | 4 | | 5.5 | | 2.5 | | 1.5 | 1 | | |  |  |
| mg85 | 6.5 | 6.5 | 6.5 | 6.5 | 6 | 7 | | 6.5 | 5.5 | | 7 | | 7 | | 7 | | 7 | | 8 | | 3.5 | | 2 | 0 | | |  |  |
| Median | 11.5 | 9.5 | 8.5 | 6.5 | 6 | 5.5 | | 6 | 5 | | 5 | | 4.5 | | 4.5 | | 4 | | 3.5 | | 2.5 | | 2 | 1 | | |  |  |

| **Table S3. Numbers of saccharin reinforcers obtained at each FR for individual subjects in Experiment 3** | | | | | | | | | | | | | | | | | | | | | | | | | |
| --- | --- | --- | --- | --- | --- | --- | --- | --- | --- | --- | --- | --- | --- | --- | --- | --- | --- | --- | --- | --- | --- | --- | --- | --- | --- |
|  | | | | | | | | | | | | | | | | | | | | | | | | | |
| Saccharin With Heroin Group | | | | | |  | |  | |  | |  | |  | |  | |  | |  | |  | |  | |
| Subject | FR1 | FR2 | FR4 | FR8 | FR12 | | FR18 | | FR24 | | FR32 | | FR40 | | FR52 | | FR64 | | FR80 | | FR96 | | FR128 | |  |
| fb1b | 114.5 | 102.5 | 77 | 71.5 | 99 | | 52 | | 50 | | 35 | | 20.5 | | 8.5 | | 1 | | 0.5 | | 0.5 | | 0 | |  |
| mb8 | 106.5 | 100 | 91.5 | 74.5 | 64 | | 48 | | 31 | | 32.5 | | 29.5 | | 18 | | 12.5 | | 9 | | 5 | | 2 | |  |
| mb4b | 44 | 17 | 6 | 5 | 1.5 | | 3 | | 0 | | 0 | | 0 | | 0 | | 0 | | 0 | | 0 | | 0 | |  |
| fb11c | 122.5 | 106 | 31 | 1.5 | 0 | | 0 | | 0 | | 0 | | 0 | | 0 | | 0 | | 0 | | 0 | | 0 | |  |
| mb28 | 113 | 95.5 | 80 | 43.5 | 64.5 | | 38 | | 16 | | 15 | | 6.5 | | 3.5 | | 1.5 | | 0.5 | | 0 | | 0 | |  |
| fb31 | 173.5 | 146 | 115.5 | 71.5 | 93 | | 60.5 | | 61 | | 31.5 | | 23 | | 7 | | 3.5 | | 3 | | 2 | | 0.5 | |  |
| fb29 | 97 | 88.5 | 71 | 59 | 60.5 | | 45 | | 39 | | 35 | | 41.5 | | 18 | | 5 | | 5 | | 5.5 | | 2 | |  |
| mb30 | 58.5 | 32 | 21 | 12 | 8.5 | | 7.5 | | 12 | | 9.5 | | 9 | | 6 | | 8 | | 5 | | 2 | | 1 | |  |
| fb23^#^ | 27.5 | 39 | 35.5 | 36 | 22 | | 12.5 | | 16 | | 4.5 | | 4 | | 3 | | 3 | | 1 | | -- | | -- | |  |
| mb24b | 111.5 | 86.5 | 45 | 29.5 | 49.5 | | 47.5 | | 24 | | 8.5 | | 7.5 | | 5 | | 1.5 | | 1.5 | | 0 | | 0 | |  |
| mb34^#^ | 25 | 23.5 | 13 | 15 | 18.5 | | 34.5 | | 20.5 | | 33 | | 28 | | 18 | | 7.5 | | 5.5 | | 2.5 | | 3 | |  |
| fb7b | 56.5 | 34.5 | 34.5 | 42 | 24.5 | | 21 | | 15.5 | | 11 | | -- | | -- | | -- | | -- | | -- | | -- | |  |
| Median | 101.75 | 87.5 | 40.25 | 39 | 37 | | 36.25 | | 18.25 | | 13 | | 9 | | 6 | | 3 | | 1.5 | | 1.25 | | 0.25 | |  |

|  | | | | | | | | | | | | | | | | | | | | | | | | | |
| --- | --- | --- | --- | --- | --- | --- | --- | --- | --- | --- | --- | --- | --- | --- | --- | --- | --- | --- | --- | --- | --- | --- | --- | --- | --- |
| Saccharin Only Group | | | | | |  | |  | |  | |  | |  | |  | |  | |  | |  | |  | |
| Subject | FR1 | FR2 | FR4 | FR8 | FR12 | | FR18 | | FR24 | | FR32 | | FR40 | | FR52 | | FR64 | | FR80 | | FR96 | | FR128 | |  |
| mb2b | 55.5 | 38.5 | 43 | 23.5 | 31.5 | | 26 | | 23 | | 17 | | 8.5 | | 11.5 | | 6.5 | | 5 | | 1.5 | | 0.5 | |  |
| fb6 | 152.5 | 76 | 57 | 61.5 | 44 | | 44 | | 23 | | 37.5 | | 11 | | 14.5 | | 5.5 | | 14.5 | | 10 | | 5 | |  |
| fb9 | 130 | 60.5 | 86 | 66 | 29.5 | | 57.5 | | 21.5 | | 36.5 | | 25 | | 15 | | 19.5 | | 2.5 | | 5 | | 2.5 | |  |
| mb10 | 90 | 65 | 56.5 | 32 | 33.5 | | 19.5 | | 8.5 | | 28 | | 16.5 | | 20 | | 9.5 | | 6 | | 7 | | 2.5 | |  |
| mb12 | 32.5 | 37 | 34 | 11.5 | 31 | | 20 | | 13.5 | | 10 | | 4 | | 4.5 | | 2 | | 3.5 | | 1.5 | | 2.5 | |  |
| fb3 | 191 | 117.5 | 111 | 77 | 78 | | 48.5 | | 34 | | 37.5 | | 25 | | 16 | | 12.5 | | 7.5 | | 6 | | 8.5 | |  |
| fb15 | 83 | 28 | 42.5 | 30 | 31 | | 33.5 | | 9 | | 23 | | 10.5 | | 11 | | 9 | | 4 | | 4.5 | | 1.5 | |  |
| mb14 | 15.5 | 7.5 | 8.5 | 4 | 6.5 | | 0 | | 0 | | 0 | | 0 | | 0 | | 0 | | 0 | | 0 | | 0 | |  |
| fb25 | 66 | 19 | 40 | 29.5 | 24 | | 43 | | 16 | | 14.5 | | 11 | | 19.5 | | 4.5 | | 38.5 | | 5 | | 2.5 | |  |
| fb27 | 57.5 | 30.5 | 43 | 28.5 | 35 | | 32.5 | | 14.5 | | 9.5 | | 9 | | 28 | | 6.5 | | 10 | | 4.5 | | 1.5 | |  |
| fb33 | 95.5 | 73 | 65.5 | 44 | 35.5 | | 17.5 | | 11 | | 6.5 | | 3.5 | | 2.5 | | 4.5 | | 2 | | 2.5 | | 1 | |  |
| mb36 | 40.5 | 55.5 | 43.5 | 32 | 42 | | 16.5 | | 21 | | 6 | | 14 | | 7 | | 8.5 | | 5 | | 4 | | 3.5 | |  |
| fb37 | 34.5 | 23.5 | 30.5 | 20.5 | 14.5 | | 15.5 | | 7 | | 6.5 | | 6.5 | | 1.5 | | 2 | | 1.5 | | 0 | | 0.5 | |  |
| Median | 66 | 38.5 | 43 | 30 | 31.5 | | 26 | | 14.5 | | 14.5 | | 10.5 | | 11.5 | | 6.5 | | 5 | | 4.5 | | 2.5 | |  |

**Figure Legends**

S1. Free-operant avoidance results. (a) Mean (± SEM) avoidance responses (i.e., responses occurring during the R-S 35-s interval of the free-operant avoidance session) per session made on the middle lever at each price of heroin on the right lever in each group. ***p* < 0.01, for the differences between the TOA Alternative group and the other two groups on the final session. (b) Mean (± SEM) escape responses (i.e., responses made during the S-S 5-s interval or during a shock) per session made on the middle lever at each price of heroin on the right lever in each group. ****p* < 0.001, for the differences between the TOA Alternative group and the other two groups on the final session. (c) Mean shocks per session delivered at the end of an R-S 35-s interval at each price of heroin in each group. (d) Mean shocks per session delivered during the S-S 5-s interval at each price of heroin in each group. **p* < 0.05, ****p* < 0.001 for group difference on final session.

S2. The top panels present scatterplots of heroin EV as a function of heroin *Q*_0_ for the three groups of Exp. 1. Circles represent female rats and triangles represent males. The bottom panels show scatterplots of the slope measure reflecting degree of substitutability/complementarity (see main text for details) as a function of heroin *Q*_0_ for the three groups of Exp. 1. The statistic *r* is the Pearson partial correlation coefficient controlling for the effect of Sex. **p* < 0.05.

S3. The top panels present scatterplots of heroin EV as a function of heroin *Q*_0_ for the two groups of Exp. 2. Circles represent female rats and triangles represent males. The bottom panels show scatterplots of the slope measure as a function of heroin *Q*_0_ in the Heroin with Saccharin group. (There was no alternative reinforcer in the Heroin Only group and so there was no slope measure.)

S4. The top panels present scatterplots of saccharin EV as a function of log saccharin *Q*_0_ for the two groups of Exp. 3. Circles represent female rats and triangles represent males. The bottom panels show scatterplots of the slope measure as a function of log saccharin *Q*_0_ for the Saccharin with Heroin group. **p* < 0.05, ** *p* < 0.01.

Figure S1

Figure S2

Figure S3

Figure S4
